# Mutations in Human αA-Crystallin/sHSP Affect Subunit Exchange Interaction with αB-Crystallin

**DOI:** 10.1371/journal.pone.0031421

**Published:** 2012-02-08

**Authors:** Ilangovan Raju, Lalita Oonthonpan, Edathara C. Abraham

**Affiliations:** Department of Biochemistry and Molecular Biology, University of Arkansas for Medical Sciences, Little Rock, Arkansas, United States of America; California State University Fullerton, United States of America

## Abstract

**Background:**

Mutation in αA-crystallin contributes to the development of congenital cataract in humans. Heterooligomerization of αA-crystallin and αB-crystallin is essential for maintaining transparency in the eye lens. The effect of congenital cataract causing mutants of αA-crystallin on subunit exchange and interaction with αB-crystallin is unknown. In the present study, interaction of the mutants of αA-crystallin with αB-crystallin was studied both *in vitro* and *in situ* by the fluorescence resonance energy transfer (FRET) technique.

**Methodology/Principal Findings:**

*In vitro* FRET technique was used to demonstrate the rates of subunit exchange of αB-wt with the following αA-crystallin mutants: R12C, R21L, R21W, R49C, R54C, and R116C. The subunit exchange rates (k values) of R21W and R116C with αB-wt decreased drastically as compared to αA-wt interacting with αB-wt. Moderately decreased k values were seen with R12C, R49C and R54C while R21L showed nearly normal k value. The interaction of αA- mutants with αB-wt was also assessed by *in situ* FRET. YFP-tagged αA mutants were co-expressed with CFP-tagged αB-wt in HeLa cells and the spectral signals were captured with a confocal microscope before and after acceptor laser photobleaching. The interaction of R21W and R116C with αB-wt was decreased nearly 50% as compared to αA-wt while the rest of the mutants showed slightly decreased interaction. Thus, there is good agreement between the *in vitro* and *in situ* FRET data.

**Conclusions/Significance:**

Structural changes occurring in these mutants, as reported earlier, could be the underlying cause for the decreased interaction with αB may contribute to development of congenital cataract.

## Introduction

α-Crystallin belongs to small heat shock (sHSP) protein family. This major lens protein in vertebrates consists of two highly homologous 20 kDa subunits, αA and αB [1], [2]. αA and αB crystallins share 57% sequence homology [3], [4] and considered as molecular chaperones by having the ability to prevent aggregation of partially unfolded proteins [5]–[9]. In its native state, α-crystallin exists as large hetero-oligomeric complexes having average molecular mass of 800 kDa [10]. α-Crystallin binds to the molten globular domains of partially unfolded proteins and the C-terminal extension and the flexible C-terminal tails of both αA and αB-crystallins are vital for ensuring solubility of the huge protein assemblies that results from this binding [11], [12]. Several αA-crystallin mutants, known to be causing congenital cataract, have been reported [13]–[19]. These are: R12C [13], R21W [14], R54C [15], R21L [16], R49C [17], G98R [18], R116C [19] and R116H [13]. In a recent study on human αA-crystallin mutants by dynamic light scattering we have reported the hydrodynamic properties including the various quaternary structural parameters such as average molar mass, mass across peak, hydrodynamic radius, and polydispersity index [20]. We have also shown, when expressed in mammalian cells, these mutants form multiple aggregates and aggresomes [21].

In the eye lens, the mutant αA can exists as homo-oligomers and hetero-oligomers associated with native αA and αB. Moreover the chaperone function of the mutated αA-crystallin will be influenced by the fact whether they exist as homooligomers or heterooligomers. There are no reports focused on subunit exchange of mutated αA-crystallins with native αA and αB-crystallins. Fluorescence resonance energy transfer (FRET) is a technique which relies on the energy transfer from an excited fluorescent donor molecule directly to an acceptor molecule through the dipole-dipole coupling mechanism [22]. This technique has long been employed as a spectroscopic ruler for measurements of nanometer-scale proximities between two fluorophores in solution [23]. Recent advances in the technique of fluorescence microscopy and digital image processing allow the application of FRET for *in situ* measurements in microscopic specimens by FRET microscopy [24], [25]. Altered interaction between αB and αA-crystallins can cause an increase in high molecular weight aggregates (HMW) and increase of HMW specifically in lens diminishes chaperone activity and leads to cataract development. Using confocal microscopy, we determined the protein-protein interaction between αB-wt and mutants of αA in HeLa cells by FRET by acceptor photobleaching method in CFP-tagged αB-wt (donor) co-expressed with YFP-tagged αA-crystallin mutants (acceptors).

## Materials and Methods

### Fluorescence probes

4-Acetamido-4′-isothiocyanatostilbene-2-2′-disulfonic acid (SITS or AIAS) from Sigma Aldrich, Lucifer yellow iodoacetamide (LYI) was purchased from Molecular Probes (Eugene, OR).

### Generation of mutants

The mutants were generated by site-directed mutagenesis and protein expression was achieved in BL21 (DE3) PLysS cells, as described earlier [26]. The expressed proteins were purified by Sephacryl S-300 HR size column chromatography, the peak fractions were collected and concentrated and re-purified by Molecular Sieve HPLC using a 600 mm×7.8 mm BIOSEPSEC 4000 column (Phenomenex). Purity of the protein was examined by SDS-PAGE.

### Labeling of human αA-wt, αB-wt and mutant αA-crystallins with different fluorescent probes

The purified αB-crystallin (3 mg/ml) (donor) was labeled with 3.2 mM of SITS in 20 mM MOPS buffer containing 100 mM NaCl (pH 7.9). The reaction mixture was incubated for ∼16 h at room temperature in the dark. The acceptor molecules, i.e., the αA-wt and its mutants (3 mg/ml) were labeled with 8.4 mM of LYI in 20 mM MOPS buffer containing 100 mM NaCl (pH 7.9) and incubated in the dark for overnight at room temperature and the LYI-labeled reactions were additionally incubated for 6 hours at 37°C. The unreacted probe was then separated from the fluorescently labeled protein on a Sephadex G25 column equilibrated with 20 mM MOPS buffer containing 100 mM NaCl (pH 7.9). The extent of labeling was determined spectrophotometrically using molar extinction co-efficients of 47,000 mol^−1^ cm^−1^ at 336 nm for SITS and 11, 000 mol^−1^ cm^−1^ for LYI at 426 nm, with a corrected protein concentration (corrected for the contribution of the dye at 280 nm).

### Measurement of subunit exchange rate *in vitro*

Fluorescence resonance energy transfer (FRET) technique was used to determine the rate of subunit exchange. The exchange reaction was initiated by mixing an equal amount (0.4 mg/ml) of SITS-labeled αB-wt with LYI-labeled αA-wt, R12C, R21L, R21W, R49C, R54C and R116C (acceptors) at 37°C in 20 mM MOPS buffer containing 100 mM NaCl (pH 7.9). At different time points of 0, 2, 4, 8, 10, 20, 45, 75, 120, 180, 240, 300 and 360 minutes, 20 µl of the reaction was removed and diluted 100× with the MOPS buffer. The emission spectrum of the sample excited at 336 nm and the intensity at 415 nm was recorded using a RF-5301PC spectrofluorometer (Shimadzu). Time dependent emission spectra were obtained at an excitation wavelength of 336 nm, and a decrease in SITS emission intensity at 415 nm and an increase in LYI emission intensity at 515 nm were determined. The rate of subunit exchange was determined by nonlinear regression analysis of the data by GraphPad Prism software.

### Vectors and cell culture reagents

The Cyan (pAmCyan1-Cl or CFP) and Yellow (pZsYellow1-Cl or YFP) expression vectors were obtained from Clontech (Palo Alto, CA), HeLa cells were purchased from ATCC (Manassas, VA), plasmid DNA extraction kits were from Qiagen (Valencia, CA) and cell culture medium, fetal bovine serum (FBS), Lipofectamine 2000, penicillin/streptomycin were from Invitrogen (CA).

### Site directed mutagenesis

To generate mutants, QuickChange site directed mutagenesis kit (Agilent Technologies Inc, CA) was used. Appropriate mutagenic primers of human αA-crystallin for the mutants, R12C, R21L, R21W, R49C, R54C and R116C were designed and used for PCR. The PCR products were amplified by using YFP-tagged αA-wt as a template DNA with the following PCR conditions: the mixture was initially denatured at 95°C for 1 min followed by 95°C for 30 sec, 60°C for 50 sec and 68°C for 5 minutes for 16 cycles and followed by overall extension at 68°C for 7 minutes. The PCR product was digested with *Dpn* I for 1 hour at 37°C and 1 µl of PCR product was transformed with XL-10 Gold competent cells. The transformants were selected on LB agar medium plates containing 50 µg/ml Kanamycin. The mutant constructs were sequenced and confirmed by DNA sequence analysis.

### Cell culture and transfection

HeLa cells (ATCC, Manassas, VA) were cultured in MEM medium (Invitrogen, CA) supplemented with 10% FBS and penicillin/streptomycin (100 µg/ml), at 37°C in 5% CO_2_ humidified chamber. About 1.0×10^5^ cells/ml were seeded into each 35 mm, sterile glass bottomed single well dishes coated with poly-d-lysine (MatTek Corporation, Ashland, MA, USA) and cultured in 2 ml of growth medium for transient transfection. The overnight adherent cells were transfected with Lipofectamine 2000 (Invitrogen, CA) according to the manufacture's protocol. Briefly, cells were co-transfected with total 2 µg/well of pAmCyan1-C1 (CFP), and/or pZsYellow1-C1 (YFP) plasmids encoding the respective crystallin gene along with 5 µl of Lipofectamine 2000. After 6 h, transfected medium was removed and replaced with fresh medium containing 10% FBS. After 48 h transfection, cells were examined in a laser scanning confocal microscope.

### *In situ* FRET analysis by live acceptor photobleaching method in HeLa cells

The acceptor photobleaching method was used in a recent report [27] to study the interaction between two proteins based on the increased intensity of donor fluorescence at the time of acceptor bleaching. In this study we used this method, the acceptor fluorescence was bleached with the help of high intensity argon laser light (100% exposure at 514 nm beam). A series of pre-bleaching and post-bleaching donor and acceptor signal collecting protocols were automated for the acquisition of pre-bleach and post-bleach images and noted the increased level of donor intensities due to de-quenching and decreased level of acceptor signal due to photo-bleaching. The increased donor (CFP) fluorescence intensity and decreased acceptor (YFP) fluorescence intensity is the sign for the occurrence of protein-protein interaction. In confocal FRET images, the homogenous regions were chosen and at least 50 regions of interest (ROI) were marked in cell images and measured the FRET. The FRET efficiency was calculated based on ten images taken from each construct examined and each experimental condition was performed 3 times and values were averaged. The FRET efficiency (E) was calculated by: E = 1- (Ipre/Ipost), where ‘Ipre’ is the pre-bleach fluorescence intensity and ‘Ipost’ is the post-bleach fluorescence intensity of the donor.

## Results

### Levels of fluorescence labeling of human αA-wt, αB-wt, and mutated αA-crystallins

Before labeling with the fluorescent probes, the proteins were purified by Sephacryl S-300 HR size column chromatography and further purified with a Molecular Sieve HPLC (Beckman Gold). The level of purity was ∼99.9% and the results of SDS-PAGE were published in our previous study [20]. The same stocks of the purified proteins were used for the present study. Although human αA-crystallin contains two cysteine residues, 1 mol of LYI was tagged with αA-wt, R21L and R21W. In cysteine modified mutants, R12C, R49C, R54C and R116C, 2 mol of LYI was tagged. SITS is known to be an amine specific reagent and 2 mol of SITS was tagged to αB-wt in all the experiments. The level of tagging with these fluorescent probes was determined by using molar extinction co-efficients as described in Methods. Differential labeling by the various probes was expected from an earlier study [26]. Before plotting Ft/F0 graph, the values were normalized as per the amount of tagging of LYI to the SITS labeled proteins. In the case of αA-wt, αA-R21L, αA-R21W, protein tagged with 1 mol of LYI was mixed with αB-wt protein tagged with 2 mol of SITS. Thus the ratio of LYI∶SITS is 1∶2 which results in a factor of 0.5 used for multiplying the relative fluorescence values at 514 nm. In the case of mutants, αA-R12C, αA-R49C, αA-R54C and αA-R116C, protein tagged with 2 mol of LYI was mixed with αB-wt protein tagged with 2 mol of SITS. Thus, the ratio is 1∶1 and the relative fluorescence values were multiplied with the factor 1.

### Rate constants of subunit exchange of αB-wt and the mutated αA-crystallins

**Figure 1:**
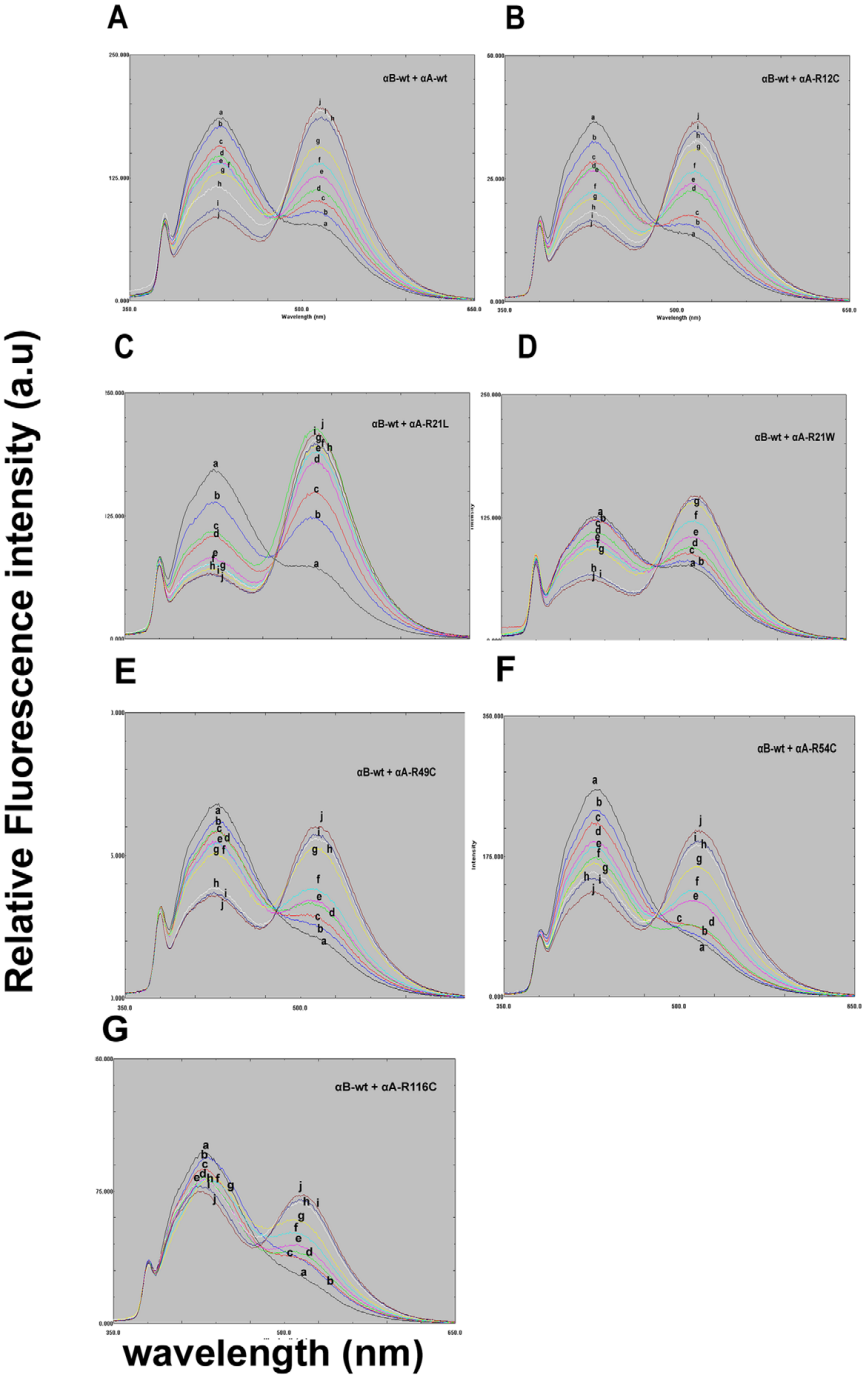
A–G: *In Vitro* FRET to demonstrate the subunit exchange between SITS-labeled αB-wt and LYI-labeled αA-wt or mutants. A representative emission spectra of αB-crystallin excited at 336 nm were recorded at 0 (a), 2 (b), 4 (c), 6 (d), 8 (e), 10 (f), 20 (g), 45 (h), 75 (i) and 120 (j) minutes after mixing of SITS-labeled αB-wt and LYI-labeled αA-wt or mutants. The decrease in fluorescence intensity at 426 nm of the SITS-labeled αB-wt protein and the concomitant increase in fluorescence intensity at 515 nm of the LYI-labeled αA-wt or mutants proteins are the indicative of energy transfer between two labeled populations. *Fig. 1A*: Time dependent spectral changes in the FRET due to subunit exchange of SITS labeled αB-wt and LYI-labeled αA-wt. *Fig. 1B*: Time dependent spectral changes in the FRET due to subunit exchange of SITS labeled αB-wt and LYI-labeled αA-R12C. *Fig. 1C*: The emission spectra of αB-crystallin excited at 336 nm were recorded at 0 (a), 2 (b), 4 (c), 6 (d), 8 (e), 10 (f), 20 (g), 45 (h), 75 (i) and 120 (j) minutes after mixing of SITS-labeled αB-wt and LYI-labeled R21L. *Fig. 1D*: The emission spectra of αB-crystallin excited at 336 nm were recorded at 0 (a), 2 (b), 4 (c), 6 (d), 8 (e), 10 (f), 20 (g), 45 (h), 75 (i) and 120 (j) minutes after mixing of SITS-labeled αB-wt and LYI-labeled R21W mutants. *Fig. 1E*: The emission spectra of αB-crystallin excited at 336 nm were recorded at 0 (a), 2 (b), 4 (c), 6 (d), 8 (e), 10 (f), 20 (g), 45 (h), 75 (i) and 120 (j) minutes after mixing of SITS-labeled αB-wt and LYI-labeled R49C. *Fig. 1F*: The emission spectra of αB-crystallin excited at 336 nm were recorded at 0 (a), 2 (b), 4 (c), 6 (d), 8 (e), 10 (f), 20 (g), 45 (h), 75 (i) and 120 (j) minutes after mixing of SITS-labeled αB-wt and LYI-labeled R54C. *Fig. 1G*: The emission spectra of αB-crystallin excited at 336 nm were recorded at 0 (a), 2 (b), 4 (c), 6 (d), 8 (e), 10 (f), 20 (g), 45 (h), 75 (i) and 120 (j) minutes after mixing of SITS-labeled αB-wt and LYI-labeled R116C. Only 10 spectral curves were shown because of RF-5301PC software is not allowing more than 10.

**Figure 2:**
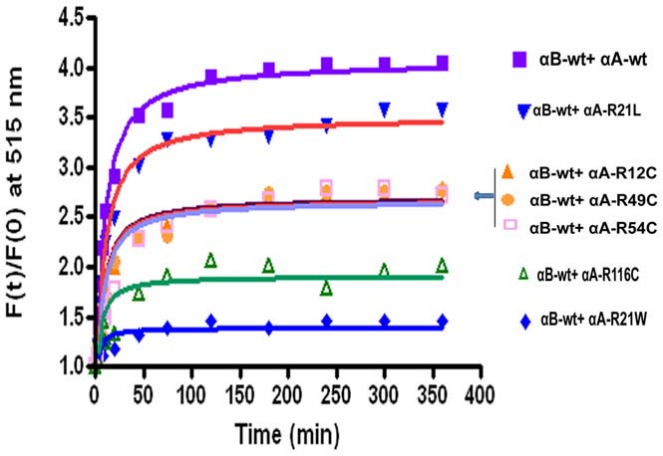
Rate of subunit exchange at acceptor energy (515 nm). Graph depicts time dependent increases in emissions intensity due to subunit exchange of αB-wt and αA-wt or mutants subunits. Increase in the relative fluorescence intensity at 515 nm due to fluorescence energy transfer from the SITS-labeled to the LYI-labeled proteins. Each curve represents the best statistical fit of the data to the exponential function of Ft/F0 = A_1_+A_2_e^−kt^. The two-tailed unpaired Student's t-test was used to determine the significance. The p value is <0.0001 for αB-wt + αA-wt Vs αB-wt + αA-R21W and αB-wt + αA-wt Vs αB-wt + αA-R116C.

The subunit exchange reaction was initiated by mixing equimolar concentration of the donor, SITS-labeled protein, with the acceptor, LYI- labeled protein. The rate of subunit exchange within heteroaggregates (wild-type or mutants of αA-crystallin+αB-wt) studied by FRET at 37°C. The time- dependant decrease in SITS emission intensity at 426 nm, and a concomitant increase in LYI fluorescence at 515 nm were indicative of energy transfer due to the proximity of the two fluorophores (Fig. 1A–G). After 2 h at 37°C, there was no change in the emission intensity due to the achievement of stable equilibrium. We have calculated the rate of subunit exchange from the increase in acceptor fluorescence intensity after taking into account differences in the levels of tagging by the various probes. Figure 2 shows the plot of Ft/F0 of LYI at 515 nm as a function of time, where Ft is the emission intensity at time t and F0 is the emission intensity at time zero. The rate constant was obtained by fitting the data to the exponential function Ft/F0 = A_1_+A_2_e^−kt^, where A_1_ and A_2_ are constants and k is the rate constant for subunit exchange. Figure 2 depicts the increase in the relative fluorescence intensity at acceptor energy (515 nm) due to fluorescence resonance energy transfer from the donor SITS-labeled protein to the LYI-labeled acceptor protein (αA-wt, αA-R12C, αA-R21L, αA-R21W, αA-R49C, αA-R54C and αA-R116C) during hetero-complex formation. αA-wt and αA-R21L show the highest fluorescence intensity, αA-R116C and αA-R21W have the lowest fluorescence whereas αA-R12C, αA-R49C and αA-R54C show intermediate range of fluorescence intensity (Table 1).

**Table 1:** Rate constants (k) of heteroaggregates of αB-wt and αA-wt and its mutants at the acceptor, 515 nm energy.

Heteroaggregates	k value (mean ± SE)
αB-wt + αA-wt	6.571×10^−4^ S^−1^±0.178
αB-wt + αA-R12C	4.369×10^−4^ S^−1^±0.234
αB-wt + αA-R21L	6.249×10^−4^ S^−1^±0.145
αB-wt + αA-R21W	1.162×10^−4^ S^−1^±0.102
αB-wt + αA-R49C	4.228×10^−4^ S^−1^±0.122
αB-wt + αA-R54C	4.441×10^−4^ S^−1^±0.142
αB-wt + αA-R116C	2.943×10^−4^ S^−1^±0.120

### Results of *in situ* FRET studies by LSM image analysis for heterologous interaction

**Figure 3:**
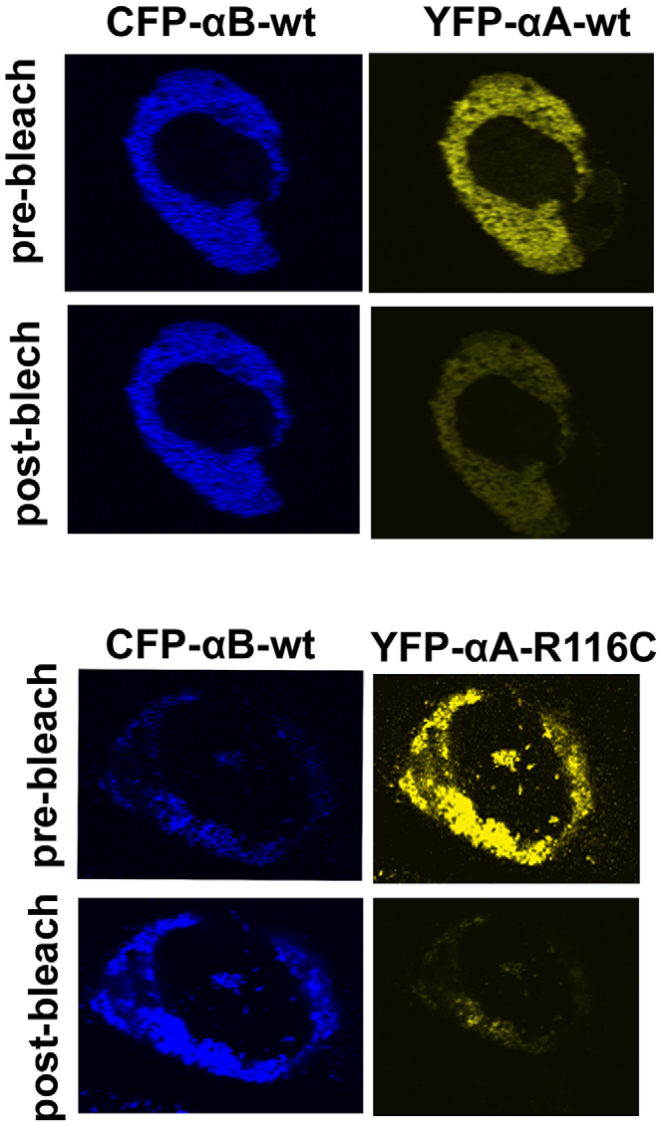
Interaction of αB-wt and αA-wt or its mutants by *in situ* FRET. Examples of the acceptor photobleaching method for determining the FRET efficiency. In this example, CFPαBwt (donor) was co-expressed with either YFPαA-wt or YFPαA-R2116C (acceptor). The acceptor fluorescence was bleached by high intensity argon laser light. This resulted in an increase in donor fluorescence intensity and a decrease in acceptor fluorescence. The actual FRET efficiency was measured from at least 50 regions of interest (ROI) in the cell images obtained in three independent experiments for each pair.

**Figure 4:**
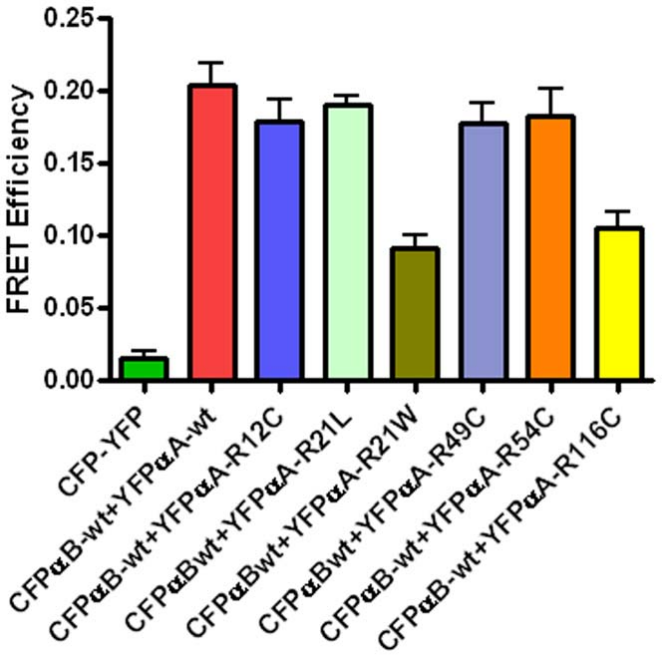
Bar diagram showing the level of FRET efficiency. FRET efficiency demonstrates the interaction between the αA and αB subunits of α-crystallin. The interaction was strong between the wild-types of αA and αB subunits. The interaction between the mutated constructs, αA-R21W and αA-R116C with αB-wt was lower to αAwt+αBwt and also lower to other mutants group. The results were expressed as mean ± SD. Two-tailed unpaired Student's t-test was used and the p value for αB-wt+αA-R21W is <0.001 and for αB-wt+αA-R116C is <0.0008 compared to αB-wt+αA-wt group.

After 48 hours transfection, cells were subjected to FRET analysis. In all experiments, 80% transfection efficiency was achieved. The acceptor photo-bleaching method was used to determine the intensities of interactions (FRET efficiency) of the mutated αA-crystallins with αBwt. It is expected that when the acceptor fluorescence is completely bleached the donor fluorescence intensity increases proportionately and this increase is considered a measure of the interaction between the two proteins. Co-expression of CFP and YFP vectors only followed by photo-bleaching of the acceptor YFP showed no increase in the donor CFP fluorescence intensity which is indicative of the lack of interactions between the vectors alone. Fig. 3 illustrates, as a representative example, photo-bleaching of YFPαA-wt (acceptor) or YFPαA-R116C (acceptor) co-expressed with CFPαB-wt (donor) leads to an increase in its fluorescence intensity. FRET efficiency values were generated from LSM images, calculated as described in ‘Methods’. These values were generated for heterologous interactions where αBwt interacts with mutated αA-crystallins (Fig. 4). As expected, negative control (vectors alone) showed very little interaction while the positive control (αBwt/αAwt) showed significant interaction. However, in αBwt/αAR21W, the FRET efficiency was ∼60% lower and αBwt/αAR116C FRET efficiency was 50% lower than CFPαBwt/YFPαAwt. FRET efficiency in αBwt/αAR21L was nearly equal to CFPαBwt/YFPαAwt. The interaction between αBwt/αAR12C, αBwt/αAR49C and αBwt/αAR54C was nearly 15% lower than in αBwt/αAwt (Fig. 4).

## Discussion

αA-crystallin/sHSP is a member of small heat shock protein family and a major structural protein in the mammalian eye lens. It exhibits chaperone function and inhibits aggregation caused by partially unfolded proteins [5]–[9]. The function of αA-crystallin is maintenance of lens transparency by preventing cataract due to protein aggregation which in turn will be dictated by the proximity of the interacting sites in the eye lens cells. The association of the mutants of αA-crystallin with αB-crystallin will be mainly determined by the strength of the interaction of the former with the later. The present study was directed toward determining the rates of the heterologous subunit exchange interactions of the mutants of αA-crystallin with native αB-crystallin. The FRET pair of fluorescent probes, SITS (AIAS) and LYI has been previously used by several investigators [28]–[30] specifically to demonstrate the rate of subunit exchange between αA and αB-crystallin proteins. LYI fluorophore was used to label cysteine residues and serving as a sulfhydryl-specific fluorophore. Labeling of cysteine residues with LYI did not appear to perturb the protein conformation [28].

It has been demonstrated that homo and heteroaggregates of αA and αB- subunits have chaperone function [5], [26], [31]. The αA-knockout studies in mice documented disruption in the stabilization of αB-crystallin, which shows that inter-subunit interaction is needed for stability and normal function of α-crystallin in the lens [32]. This study also showed that lenticular complexes of αB-crystallin formed in the absence of α-crystallin are mostly insoluble and are present as large and dense cytoplasmic inclusion bodies. It clearly indicates that heteroaggregates of αA- and αB-crystallins are the functional unit essential for maintaining the lens transparency.

In this study, we have also investigated the protein-protein interaction of αB-wt and αA mutants by *in situ* FRET assay in mammalian cells (HeLa cells) by laser scanning confocal microscopy. Protein-protein interaction of αA-crystallin mutants with αB-wt crystallin has not been investigated before and this study not only provides information on interactions between these two proteins, but also when such interactions are modified. The interaction of mutants, R21W and R116C, with αB-crystallin was significantly decreased in both *in vitro* and *in situ* FRET assays and this may explain why αB-crystallin is unable to fully protect them from forming insoluble aggregated proteins due to weaker interactions. Disruption of protein-protein interaction between αA-mutants and αB-crystallin suggests that mutations in αA-crystallin affect the interaction sites by unfolding which exposes the buried hydrophobic sites. Based on this and an earlier study [21], we propose that a mutant αA-crystallin exists in two forms, αB-crystallin bound heterooligomer and unbound homooligomer. The k values (Table 1) for the subunit interaction will be a major factor in the existence of heterooligomers vs homooligomers. For instance, both αA-R21W and αA-R116C, with the lowest k values will show preference to be in the homogligomeric form for subsequent formation high molecular mass proteins [20] and degraded through ubiquitin-proteosome pathway [21].
